# A natural language processing model for supporting sustainable development goals: translating semantics, visualizing nexus, and connecting stakeholders

**DOI:** 10.1007/s11625-022-01093-3

**Published:** 2022-02-04

**Authors:** Takanori Matsui, Kanoko Suzuki, Kyota Ando, Yuya Kitai, Chihiro Haga, Naoki Masuhara, Shun Kawakubo

**Affiliations:** 1 Division of Sustainable Energy and Environmental Engineering, Graduate School of Engineering, Osaka University, Yamadaoka 2-1, Suita, Osaka 565-0871 Japan; 2 School of Human Science and Environment, University of Hyogo, Shinzaike-honcho 1-1-12, Himeji, Hyogo 670-0092 Japan; 3 Department of Architecture, Faculty of Engineering and Design, Hosei University, 2-33 Ichigayatamachi, Shinjuku, Tokyo 162-0843 Japan

**Keywords:** Sustainable development goals, Nexus and interlinkages, Matchmaking stakeholders, Artificial intelligence technology, Text classification, BERT model

## Abstract

Sharing successful practices with other stakeholders is important for achieving SDGs. In this study, with a deep-learning natural language processing model, bidirectional encoder representations from transformers (BERT), the authors aimed to build (1) a classifier that enables semantic mapping of practices and issues in the SDGs context, (2) a visualizing method of SDGs nexus based on co-occurrence of goals (3) a matchmaking process between local issues and initiatives that may embody solutions. A data frame was built using documents published by official organizations and multi-labels corresponding to SDGs. A pretrained Japanese BERT model was fine-tuned on a multi-label text classification task, while nested cross-validation was conducted to optimize the hyperparameters and estimate cross-validation accuracy. A system was then developed to visualize the co-occurrence of SDGs and to couple the stakeholders by evaluating embedded vectors of local challenges and solutions. The paper concludes with a discussion of four future perspectives to improve the natural language processing system. This intelligent information system is expected to help stakeholders take action to achieve the sustainable development goals.

**Supplementary Information:**

The online version contains supplementary material available at 10.1007/s11625-022-01093-3.

## Introduction

The decade ending in 2030 is the Decade of Action (United Nations 2020). 2030 is the milestone year of limiting global warming to well below 1.5° (UNFCCC 2015) and of “living in harmony with nature” in 2050 (UNCBD 2021), so reaching the goals requires hastening the related activities. Various platforms have been proposed and developed to support information gathering and knowledge-sharing platforms to promote the sustainable development goals (SDGs) (Nilsson et al. 2018). Further development is expected to enable transactions and innovation of the most advanced SDGs actions and research under digital platforms to reach the stated goals (Bonina et al. 2021).

Since SDGs require multistakeholder partnerships, knowledge platforms must be established at both multiscale and multisector levels. At the global scale, the sustainable development knowledge platform (UNDESA 2021) is representative, while (Sustainable Development Solutions Network 2021) created a tracking and monitoring platform to share government sectors’ progress and maintain accountability. For the business sector, SDG compass (Global Reporting Initiative, UN Global Compact, and WBCSD 2015) provided practical information and tools, and (WBCSD 2021) also offered the SDG Essentials for Business, a learning suite for corporate SDGs activities. In the academic sector, Higher Education Sustainability Initiative (United Nations 2012) is a networking platform for over 300 universities from around the world and the Technology Facilitation Mechanism (UNDESA and UNOICT 2020) has a platform for sharing scientific and technological suggestions, ideas, and solutions for enhancing SDG activities. At the local scale, the Local 2030 (United Nations 2017) support now municipalities’ in monitoring, evaluating, and reviewing their SDGs progress, and the Voluntary Local Review Lab (Institute of Global Environment Strategy 2019) networks the municipalities released the Voluntary Local Review (VLR) reports. In Japan, the Cabinet launched the flagship “Regional Revitalization Public–Private Partnership Platform” to promote domestic SDGs activities and revitalize local areas on a national scale (Cabinet Office Japan 2020). Japan’s Ministry of Foreign Affairs manages the “JAPAN SDGs Action platform” which is a best-practice database of SDGs activities from all sectors (Ministry of Foreign Affairs Japan 2019). The private sector also launched an open innovation platform named “SHIP (SDGs Holistic Innovation Platform)” to share technologies and know-how (Japan Innovation Network and UNDP 2021).

Local governance promotion consistent with global and national scales is very important (Oosterhof 2018) and enhances the mainstreaming of SDGs (Masuda et al. 2021). It is against this background that the authors built the “Local SDGs platform”—a SDGs action supporting system operating on a local scale in Japan since 2017 to the present (Kawakubo 2018). The platform covers 1740 municipalities in Japan and facilitates progress analysis of SDGs in each municipality by using localized SDGs indicators (Kawakubo and Murakami 2020; Cabinet Office Japan 2019). These indicators were developed by adapting the UN’s 244 SDGs indicators (UNSTATS 2017) to the Japanese context. At the same time, municipalities can use the platform to present their valuable experience as narratives. All this enables the municipalities to check and review SDGs progress quantitatively as key performance indicators and share their solutions with their peers. Based on this history, the authors launched a new advanced SDGs communication platform—“Platform Clover” (Sustainable Transition 2021). This expanded the reach beyond just municipalities to all stakeholders. Platform Clover aims to be a base for SDG17 partnerships, providing bottom-up matching that incorporates a variety of goals, missions, experiences, technology, and knowledge.

Artificial intelligence (AI) technology is useful for achieving SDGs (Vinuesa et al. 2020), so AI technology will be utilized to upgrade the semantic analyzing functions of Platform Clover. Our focus will be on these core functions: (1) semantic SDG mapping, (2) SDGs interlinkages and nexus visualization, and (3) stakeholder interpretation and matchmaking. The literature review is below.

### Semantic SDG mapping

People with limited knowledge of SDGs have difficulty in translating and mapping their local challenges and activities on to the broader SDGs context. The mapping support function by AI technology should help in this area (Varshney and Mojsilovic 2019). However, this research is still ongoing. The most advanced research can be found on the Open Source SDG (OSDG) project (Pukelis et al. 2020). The OSDG developed a holistic SDGs ontology by coupling a conventional SDGs ontology and a SDGs multi-label classification system by linking a regression model and a topic model. As for machine learning studies, (Pincet et al. 2019) implemented a single-label classification task with a tree-based decision algorithm, while (Sciandra et al. 2020) employed a Gradient Boosting Decision Tree to binarily classify SDGs related tweets on Twitter into an information class or an action class. (Nugroho et al. 2020) used a naïve Bayes classifier to divide news articles into related SDGs and (ElAlfy et al. 2020) classified Corporate Social Responsibility and sustainability reports by FastText algorithm. In Japan, (Koyamada 2019) mapped the policy briefs produced by the Japanese Science Council to relevant SDGs. All this suggests that the demand for technology to link social challenges, policy, and science is quite high.

### SDGs nexus visualizing

As emphasized in the preamble to the 2030 agenda, SDGs must be attained by ensuring interlinkages of SDGs and targets. However, the interlinkages among SDGs are very complex and wicked (Bowen et al. 2017), with the importance of both synergy and trade-offs in achieving global optimization repeatedly pointed out (Allen et al. 2018; Del Río Castro et al. 2021; Kroll et al. 2019). These interlinkages and interactions are also referred to as the ”SDGs nexus” (Liu et al. 2018), and this paper employs the word “nexus” in the same way as “interlinkages” in this paper. The visualization of SDGs nexus enables science-based support for effective allocation and distribution of resources and a proactive design of synergy and trade-offs in policy making. For a decade, the authors also had challenged qualitative and quantitative nexus assessments of Japanese prefectural scale (Kumazawa et al. 2009, 2014; Matsui et al. 2019; Masuhara et al. 2019). Recently, SDGs nexus research gains attention from knowledge driven to data driven approach: an integrated research that summarizes key papers (Scharlemann et al. 2020; Alcamo et al. 2020), empirical studies that identify the SDGs interlinkage from VNR’s documents and statistics (Zanten and Tulder 2021; Tosun and Leininger 2017; Sebestyén et al. 2019; Bali Swain and Ranganathan 2021; Fonseca et al. 2020), model-based studies delineate the synergistic or trade-off interactions using the Integrated Assessment Models (van Soest et al. 2019), text mining and network research from documents (Sebestyén et al. 2020), machine learning applications to predict SDG interlinkages (Requejo-Castro et al. 2020), Causality Analysis interconnected SDG factors (Dörgő et al. 2018), a visualizer development of SDG interlinkage (Zhou et al. 2019).

### Connecting and matchmaking for collaboration, partnership, and cooperation

The promotion of SDG 17 partnership for the goals is expected to expedite the matching of challenges and problems to know-how and solutions among various stakeholders (Chon et al. 2018; Richards-Kennedy and St Brice 2018; Saric et al. 2019). However, since such matching is still at the proof-of-concept stage, research is sparse. Early studies have only examined the definition of collaboration, partnership, and cooperation in the SDGs context (Stott and Murphy 2020) and guiding collaboration design and governance for contributing SDGs in the business sector (Vazquez-Brust et al. 2020). In the Japanese context, (Cabinet Office Japan 2020) has conducted manual matchmaking exercise of stakeholders, but this has proven a time-consuming task. At a practical level, the UNEP has attempted to apply association rule learning to smart matchmaking of stakeholder (International Telecommunication Union 2020). These matching support systems elaborate the opportunity for all stakeholders to discover potential innovations.

Against this background, this study aims to build a natural language processing system with three functions; (1) a text classifier to map challenges and activities to SDGs context at the goal level; (2) an interlinkage visualizer of the SDGs nexus; (3) semantic matchmaking between local challenges and potential solutions from a variety of stakeholders.

## Methodology

**Fig. 1:**
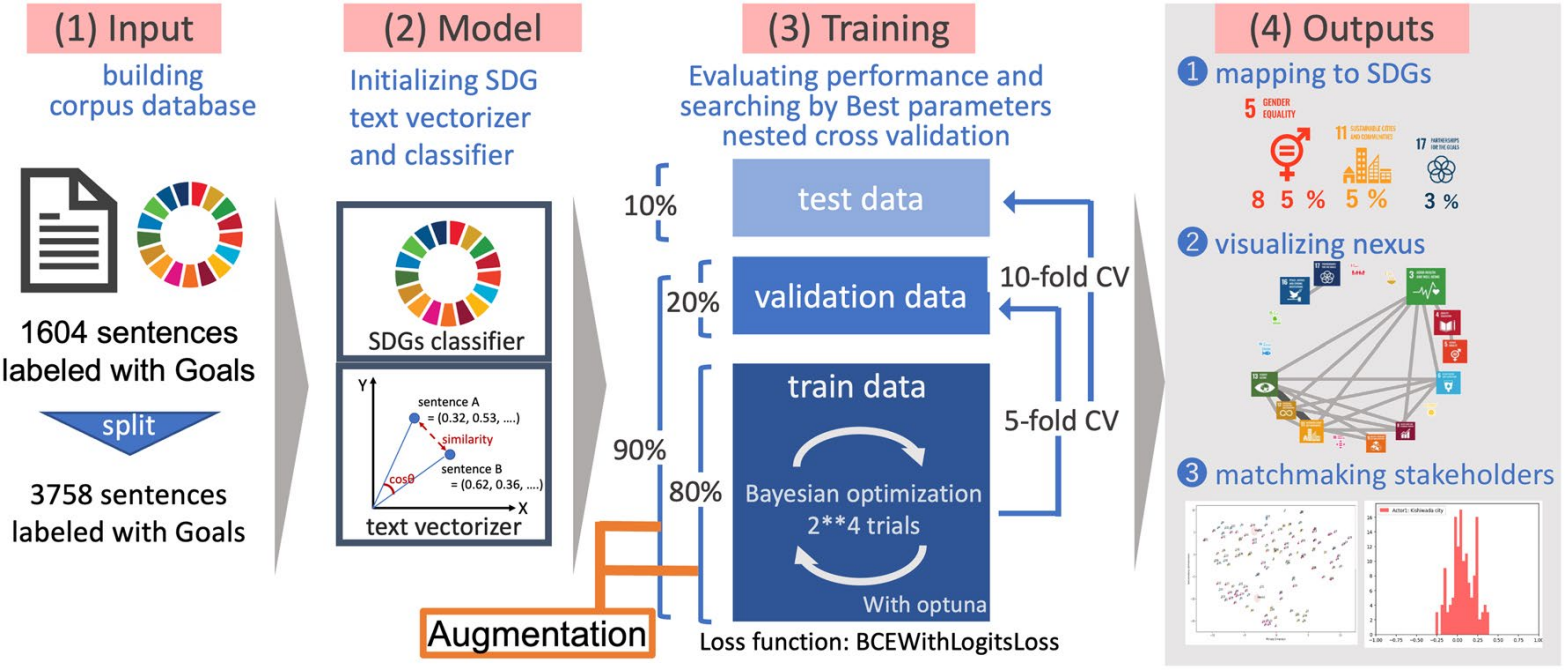
An overall structure in building corpus database, defining the model structure, training best model, and applying the best model to solutions. The numbers in each process correspond to section numbers

The comprehensive analytical framework is shown in Fig. 1. The detailed process of (1) building the corpus database for model training, (2) initializing natural language processing model, (3) training and validating the model, (4) applying the model to SDGs mapping, nexus visualizing, and stakeholder matchmaking are shown below.

### Building SDGs corpus database for model training

Japanese documents that explicitly refer to SDG’s goals, targets, and indicators were collected, along with explanatory addendums. This was also done with documents from the United Nations, the Japanese government, and the private sector. The 41 documents are listed in Supplementary material 1. The documents were checked manually and sentences related to the SDGs were extracted. Table 1 shows the samples. This is the initial corpus (*N* = 1604) and includes both text and 17-dimensional multi-label data. If a sentence is related to SDG3, 5, 10, and 15, the 17-dimensional multihot vector is [0, 0, 1, 0, 1, 0, 0, 0, 0, 1, 0, 0, 0, 0, 1, 0, 0]. The mean characters/sentence and token/sentence was 1303.5 and 780.9, respectively, in the initial corpus. However, the BERT model (Devlin et al. 2019), which is a natural language processing model used for this research (explained below), originally has the specification that the maximum length of the input length of the tokens is =  < 512. The mean token/sentence exceeded the acceptable vector length of the BERT model, so the sentences were divided to avoid exceeding the 512 token limitation even if all the characters were individually tokenized into a character (e.g., sentences with 1,024 characters was divided into two sentences with 512 characters with same the 17-dimensional multihot vector). As the result, the SDGs corpus database for training was increased to (*N* = 3758).

**Table 1:** Samples of corpus database

ID	SDGs multi label	Original sentences in Japanese	Translated text in English
1	[0, 0, 0, 0, 1, 0, 0, 1, 0, 0, 0, 0, 0, 0, 0, 1, 0]	学校の授業は「持続可能な開発目標」を実現させる大きな力になる!、岐阜県のある中学校では、中学校2年生の英語の学習で "Landmines and AkiRa"を学んだ際、日本ユニセフ協会に出前授業を依頼し、地雷についての授業を行いました。生徒たちは地雷や不発弾のレプリカを実際に見て手に取り、内戦以後も地雷や不発弾によって死傷するカンボジアの多くの子どもたちのこと、自分たちができることに ついて深く考えさせられました。その学習がきっかけとなり、のちに生徒たちは募金活動を行い、集まった募金をユニセフに贈呈するなど、世界の子どもが直面している問題について学ぶだけでなく、自分たちに何ができるのかを考え、行動に繋げることができました。日本ユニセフ協会では、地雷の木製レプリカセット地雷教育用のポスタ指導用のパワーポイントが入った学習キットを貸し出しています。(length = 378)	School lessons will be a great force to achieve the “Sustainable development goals”! At a junior high school in Gifu prefecture, when I learned “Landmines and Aki Ra” in the second year of junior high school, I asked the Japan Committee for UNICEF to give a class about land mines. The students actually saw and picked up replicas of landmines and unexploded ordnance, and were made to think deeply about many Cambodian children who were killed or injured by landmines and unexploded ordnance even after the civil war, and what they could do. The learning triggered the students to raise funds and donate the collected funds to UNICEF, not only to learn about the problems facing children around the world, but also to learn what they can do. I was able to think and act. The Japan Committee for UNICEF rents out a wooden replica set of landmines, a power for teaching posters for landmine education, and a learning kit containing her points. (length = 942)
2	[0, 0, 0, 0, 0, 0, 0, 0, 0, 0, 0, 0, 1, 0, 0, 0, 0]	気候変動債、グリーンボンド、その他の債券や証券などを通じ、気候変動リスクの軽減、気候変動へのレジリエンスおよび気候変動への適応のために投資や資金提供を行う。国および地域の自然災害保険スキームの補償範囲を拡大する。保険引受業務、投資分析および意思決定に気候変動リスクを組み入れる。。設定した目標に照らして気候変動によるリスクを測定、軽減、報告し、気候変動に立ち向かうための対策を進展させると同時に、産業セクター全体の報告の透明性と一貫性のレベルを継続的に向上させていく。(length = 234)	Invest and fund climate change risk mitigation, climate change resilience and climate change adaptation through climate change bonds, green bonds, and other bonds and securities. Expand coverage of national and regional natural disaster insurance schemes. Incorporate climate change risk into underwriting, investment analysis, and decision making. Measure, mitigate and report on the risks of climate change against the goals set and develop measures to combat climate change while continually improving the level of transparency and consistency of reporting across the industrial sector. (length = 590)
3	[0, 0, 1, 0, 0, 0, 0, 0, 0, 0, 0, 0, 0, 0, 0, 0, 1]	「新たなスポーツ施策の振興及びスポーツを活用した地域活性化」。地域活性化、健康福祉、観光客の誘致・地域PR (インバウンドを含む) 、情報化 (ICT・IoT・AIの利活用等) 。社団法人・財団法人、NPO・NGO、大学・教育機関・研究機関・国機関等、宿泊・飲食サービス業、運輸・通信業、サービス業。連携のイメージは、平塚海洋エネルギー研究会のウェブをご覧ください。情報収集・共有及び意見交換等を行いたい。自動車関連や化学系の大手・中小の工場、研究所が多数立地。商業、農業、漁業が一定規模ある。。東京大学生産技術研究所林研究室との共同研究(3年間)を計画している。テレワークの増加やCO_2_排出削減の世界的な動きに対応するため、国産技術での再生可能エネルギーの普及を目指しています。(length = 336)	“Promotion of new sports measures and regional revitalization utilizing sports.” Regional revitalization, health and welfare, attracting tourists/regional PR (including inbound), computerization (utilization of ICT/IoT/AI, etc.). Incorporated associations/foundations, NPOs/NGOs, universities/educational institutions/research institutes/national institutions, etc., accommodation/food service industry, transportation/communication industry, service industry. For an image of the collaboration, please see the website of the Hiratsuka Marine Energy Study Group. I would like to collect and share information and exchange opinions. There are many large, small, and medium-sized factories and research institutes in the automobile and chemical fields. There is a certain scale of commerce, agriculture, and fishing. We are planning a joint research (3 years) with Hayashi Laboratory, Institute of Industrial Science, University of Tokyo. We are aiming to popularize renewable energy using domestic technology in order to respond to the increase in telework and the global movement to reduce CO_2_ emissions. (length = 1104)
4	[0, 0, 0, 0, 0, 1, 0, 1, 0, 0, 0, 1, 0, 0, 0, 0, 1]	「湯の丸高原天然水のブランド化に向けた取り組み」。地域活性、観光客の誘致・地域PR (インバウンドを含む) 、その他 (地域資源の有効活用、水道事業の経営安定化) 。宿泊・飲食サービス業、卸売・小売業、飲食店、電気・ガス・水道・熱供給業水販売戦略を有し、販売実績のある事業者を希望する。・湯の丸水源の天然水の販売のためのコンセプトや販売先へ"強み”や他地域との差別化の検討・具体的な販路開拓。情報収集・共有及び意見交換等を行いたい。自動車関連や化学系の大手・中小の工場、研究所が多数立地。商業、農業、漁業が一定規模ある。令和3年度の予算計上を検討している。テレワークの増加やCO_2_排出削減の世界的な動きに対応するため、国産技術での再生可能エネルギーの普及を目指しています。(length = 332)	“Efforts to brand Yunomaru Kogen natural water.” Regional revitalization, attracting tourists/regional PR (including inbound), etc. (effective utilization of regional resources, stabilization of water supply business management). Accommodation/restaurant service industry, wholesale/retail industry, restaurant, electricity/gas/water/heat supply industry we would like to have a business with a water sales strategy and a sales record.・ Consideration of “strengths” and differentiation from other regions to the concept and sales destinations for selling natural water from Yunomaru water source.・ Development of specific sales channels. I would like to collect and share information and exchange opinions. There are many large, small, and medium-sized factories and research institutes in the automobile and chemical fields. There is a certain scale of commerce, agriculture, and fishing. We are considering budgeting for the third year of Reiwa. We are aiming to popularize renewable energy using domestic technology in order to respond to the increase in telework and the global movement to reduce CO2 emissions. (length = 1116)
5	[1, 0, 0, 0, 0, 0, 0, 0, 0, 0, 0, 1, 0, 0, 0, 0, 0]	廃棄物処理 (再生燃料製造) 、環境修復事業。SDGs貢献に向けた取り組みの概要。【海外事業のきっかけは、Team-E KANSAI】。海外事業は、Team-E KANSAI (海外展開支援機関、経済団体、自治体、企業等で構成) の歩みとともにある。。Team-E KANSAIのミッションなどで、アジア各国を訪問し、社会課題とともに、ビジネスチャンスがあることがわかった。。【海外と日本をつなぎ、さまざまな社会課題を解決】。マレーシア、インドネシアは、世界の80%以上のパームオイルを生産している。。それに伴う未利用物、排水が社会問題になっている。。そこで、未利用物であるパーム房 (EFB) を自社技術で、木質ペレットに加工し、発電所燃料として利用し、その燃焼灰はセメント工場でセメント原料にしている。このように、アジアの環境問題の解決は、他の社会課題の解決にもつながっている。(length = 385)	Waste treatment (recycled fuel production), environmental restoration business. Outline of efforts to contribute to the SDGs. [Team-E KANSAI was the catalyst for overseas business]. The overseas business is in line with the progress of Team-E KANSAI (composed of overseas expansion support organizations, economic organizations, local governments, companies, etc.). I visited Asian countries on the mission of Team-E KANSAI and found out that there are business opportunities as well as social issues. [Connecting overseas and Japan to solve various social issues]. Malaysia and Indonesia produce more than 80% of the world’s palm oil. Along with this, unused materials and wastewater have become a social problem. Therefore, the unused palm bunch (EFB) is processed into wood pellets using our own technology and used as fuel for power plants, and the combustion ash is used as a raw material for cement at a cement factory. In this way, the solution of environmental problems in Asia has led to the solution of other social issues. (length = 1034)

Note: The column 1 is sentence id, column 2 is the 17-dimensional multi hot vectors which mean the correspondence to the SDGs (correspondent 1, not correspondent 0), column 3 and column 4 are the original Japanese sentences and the English sentences translated by Google translate. The original Japanese sentences were used for the model training and validation

### Initializing the natural language processing model

The BERT model—”Bidirectional Encoder Representations from Transformers” developed by (Devlin et al. 2019)—was applied as the natural language processing model to learn the corpus. BERT can be applied to various tasks of natural language processing and performed impressively when measured by General Language Understanding Evaluation (known as GLUE) (Wang et al. 2019), which is the standard benchmark task in the natural language processing research field. And many experiments showed the superiority of BERT task against other machine learning algorithms in text classification (González-Carvajal and Garrido-Merchán 2021). The transformers library (Wolf et al. 2020) (== 3.0.2) developed by Hugging Face (Hugging Face 2016), which is the natural language processing suites implemented on the deep-learning framework (Jax, Pytorch, and Tensorflow), was utilized. This Japanese BERT model pretrained by Japanese Wikipedia on Pytorch framework (== 1.6.0) released by Tohoku university Japan (Inui Laboratory 2019; Suzuki 2021) was adopted. The base model of the Tohoku-BERT (cl-tohoku/bert-base-japanese-whole-word-masking) was used. The Tohoku-BERT employs Japanese morphological analysis Mecab (MeCab 2006) with the ipadic dictionary (Asahara and Matsumoto 2003) (== 2.1.2) and WordPiece algorithm (Sennrich et al. 2015) for the tokenizer. And the BERT model was rebuilt for the multi-label classification task. This was further fine-tuned through the model learning the SDGs corpus database. The original Tohoku-BERT model architecture consists of 12 attention layers with 12 attention multi-heads, and the input for the model is =  < 512 tokens from sentences and the output is 768-dimensional vectors by the transformer encoders. Therefore, the input token length was fixed to 512 and a fully connected layer with sigmoid activation function was added after the transformer encoder, allowing input to 768-dimensional vector of the CLS token and output with a 17-dimensional vector. The model was initialized to predict the probabilities of the input sentence belonging to each SDGs.

### Training and evaluation of the model

In the process of model training, nested cross-validation (CV) (Varma and Simon 2006) was conducted to train the prediction model with the best combination of hyperparameters and to estimate the expected cross-validation loss at the same time. The inner CV (innerCV) detects the optimum combination of hyperparameters while the outer CV (outerCV) evaluates the expected classification performance. Tenfold was set for outerCV and fivefold for innerCV for the nested CV due to time constraints. This phase involved text data augmentation to the training data frame in each outerCV and innerCV. In the text augmentation, the training data frame was copied and the ten percent tokens included in the copy were replaced by a random synonym predicted by WordNet (Miller 1995) implemented in the nltk library (NLTK 2021). Furthermore, ten percent tokens in the copied data frame were then randomly deleted and the copy was merged with the original data frame.

In the fine-tuning process, the pretrained model parameters of all attention heads in the 1st to11th layers were frozen and the 12 attention heads in the last 12th layer and the final fully connected layers were set as trainable. This operation is expected to facilitate compatibility between the common sense from Wikipedia and the idea of SDGs specific context. The binary cross-entropy with logit loss was set as the loss function for the training and Adam (Kingma and Ba 2017) set as the optimization algorithm of the model parameter. The Bayesian optimization library Optuna (== 1.3.0) (Akiba et al. 2019) was used to search the optimum combination of the batch size (ranged from 2^2^ to 2^5^) for training, the learning rates of the transformer encoder, and the fully connected layers (bath ranged from 10^−5^ to 10^−2^). The objective function was the mean loss of each innerCV, with the trial and epoch number set to 2^4^. These search ranges and the numbers of trials were determined with reference to the trial and error and time limitation in the pretest stage.

The optimal hyperparameters detected by the innerCV were set in each outerCV and the expected performance was evaluated based on the aggregated performance of outerCV. The precision metrics were accepted, along with the recall and f1-score for the evaluation of classification performance.

1 $$ {\text{Precision}}_{i} = \frac{{TP_{i} }}{{TP_{i} + FP_{i} }} $$

2 $$ {\text{Recall}}_{i} = \frac{{TP_{i} }}{{TP_{i} + FN_{i} }} $$

3 $$ F1\quad {\text{score}}_{i} = \frac{{2 \times {\text{Precision}}_{i} \times {\text{Recall}}_{i} }}{{{\text{Precision}}_{i} + {\text{Recall}}_{i} }} $$

where, *TP* (true positive) and *TN* (true negative) are the numbers of the correct prediction to positive and negative samples, respectively. And conversely *FN* (false negative) and *FP* (false positive) are the numbers of the incorrect prediction to positive and negative samples, respectively. The precision *i* is the ratio of samples predicted to class *i* that actually belonged to said class (Eq. 1). The recall is the ratio of correctly identified spectrogram numbers to the total spectrogram numbers of class *i* (Eq. 2). The F1 score is the harmonic mean value of precision and recall of class *i* (Eq. 3).

Lastly, the best model was trained for SDGs mapping, nexus visualization, and stakeholder matchmaking by setting the mean of the optimal hyperparameter set obtained in each outerCV to the best hyperparameters and the epoch to 2^5^ times.

### Application: SDGs mapping, nexus visualizing, and stakeholder matchmaking

This model was used in three applications. First, in the evaluation of SDGs mapping performance, an unknown text, which was not used for the training, was inputted. The BERT model can produce three outputs in the prediction process; semantic vector of the unknown text; membership probability distribution to SDGs; attention weight to the tokens contributing to the classification decision. Hereby the SDG related to the unknown text was predicted quantitatively and the validity of the mapped SDGs and attention to the tokens were qualitatively evaluated by interpreting the semantic features of the unknown text.

As an application case of the text classification model, the Inventory of Business Indicators released from SDG compass (Global Reporting Initiative, UN Global Compact, and WBCSD 2015) (*N* = 1479, translated in Japanese) was input, and the SDGs related to each indicator were predicted in multi-label format. The co-occurrence of predicted SDGs was analyzed and the network structure as a plausible SDGs nexus visualized.

For an application case of matchmaking of stakeholders, the stakeholder’s database released by (Cabinet Office Japan 2020) was used. (Cabinet Office Japan 2020) regularly holds the matchmaking and networking event beyond the industry, government, academia, and private sectors in their platform and manually matchmake the potential collaborations. This phase simulates the matchmaking application between needs and resources of SDGs by using the semantic vectorizing function and the dimension reduction algorithm.

## Results

### Performance of multi-label classification

The corpus for the training was (*N* = 2483), and the mean, maximum, and minimum token/sentence were, respectively, 237.8, 368, and 2. The number of cumulative and unique tokens were 893,739 and 12,290. The Tohoku-Japanese-BERT has 32,000 vocabularies, so the SDGs’ semantic space was defined at 38.4% (12,290/32,000) of the vocabularies. Unknown token that was not in the vocabulary of Tohoku-Japanese-BERT token was not included in the training corpus. The distribution of the numbers of labels by SDGs was not uniform, with the maximum being 1,946 for SDG 08: “decent work” and “economic growth.” The minimum was 773 for SDG 06: “clean water and sanitation.”

The performance of the nested CV was shown in Table 2. It took 265 h for the training on Nvidia Graphical Processing Unit (GPU) Quadro GV100 32 GB with CUDA 10.1 cuDNN 7.6.5. The overall precision, recall, macro-f1-score were, respectively, 0.95, 0.94, and 0.95, which achieved a high cross-validation performance. The recall and precision by SDGs were over 0.90 in all SDGs, with macro f1-scores ranging from 0.92 to 0.97. The mean and standard error of the best hyperparameters obtained by tenfold outerCV, the batch size, the learning rate of the BERT encoder, and the fully connected layer were, respectively, 2^3.7^ (0.3), 1.1 × 10^−4^ (0.8 × 10^−4^), and 1.3 × 10^−4^ (0.4 × 10^−4^).

**Table 2:**
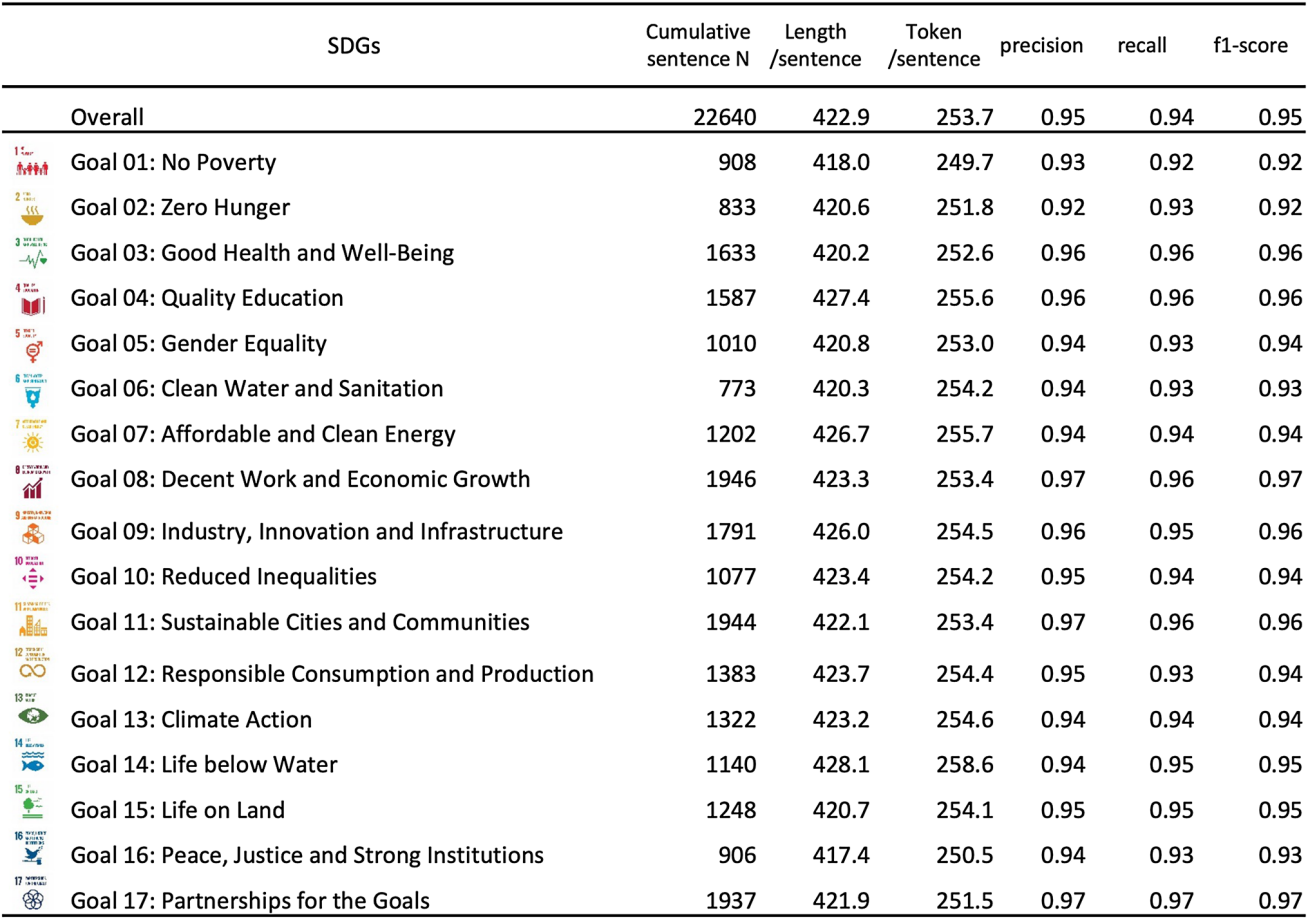
Performance of nested cross-validation

In summary, the classification performance can be regarded as excellent. However, these stats indicate that the introduction of richer corpora and weighted loss against the imbalanced class distribution approach, or an increase in the number of the Bayesian optimizations, may improve performance.

### SDGs semantic mapping

**Fig. 2:**
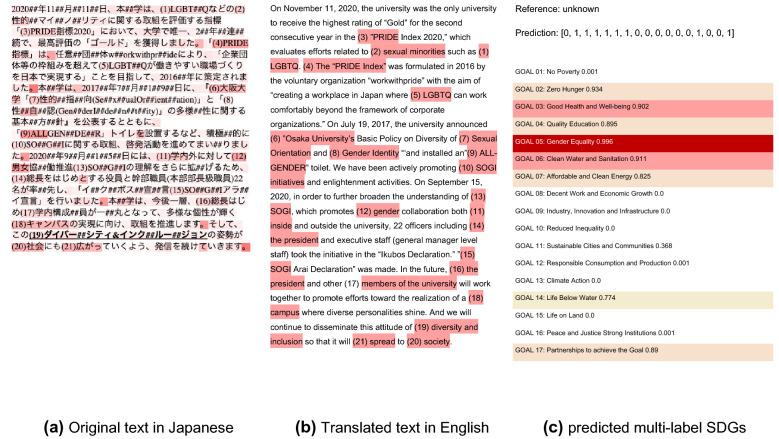
SDGs mapping by text classification and attention visualization: a sample of SDG 5 Gender equality. Note: The red markers in the panel **a** and **b** mean the BERT model pays attention to the tokens. The color intensity is consistent with the level of attention. And the red markers in panel **c** mean the high probability of the prediction. The ## in the panel **a** indicates the sub word division by WordPiese algorithm

A trial of SDGs mapping by multi-label text classification and attention visualization against the unknown data is shown in Fig. 2. The text in Fig. 2 (a) is an original Japanese news article published when Osaka University won an award for its policies to promote equality for sexual minorities (Osaka University Center for Gender Equality Promotion 2021), and Fig. 2 (b) is English translation using Google translate (Google 2021). This article was not included in the training corpus, so it is unknown data to the model. Figure 2 (c) shows the predicted probability vectors by SDGs in multi-label format related to the input article, with the tokens in red color intensity Fig. 2 (a) being the attention weights that the model referenced in the prediction process as key tokens [the red in Fig. 2 (b) were manually added to the highly highlighted tokens].

The top probability of the prediction was SDG 05: gender equality at 99.6%, which sounds appropriate in terms of the description. The tokens with high attention weights were [diversity, gender, LGBTQ (Lesbian, Gay, Bisexual, Transgender and Queer), sexual minority, SOGI (Sexual Orientation and Gender Identity)] and these categories were robustly connected to gender equality and diversity. The main topic was the introduction of all-gender toilets in cooperation with all of the Osaka University members, so the prediction of SDG03 (good health and well-being), SDG04 (Quality education), SDG 06 (clean water and sanitation), and SDG 17 (partnership for the goals) also fit. The high probabilities of SDG 02 (zero hunger), SDG 07 (affordable and clean energy) implicitly may propose some strong nexus hypothesis between gender activities, reducing hunger, and renewable energy implementation. (This aspect is discussed further in the SDGs nexus section below.)

On the other hand, the process also highlights a specifically Japanese language problem in token (19) [インクルージョン&ダイバーシティ] in Japanese in Fig. 2 (a) and [inclusion & diversity] in English in Fig. 2 (b). Japanese uses a mixture of four writing systems, Chinese characters, Hiragana, Katakana, and Alphabet. The token “diversity” can be written in {Chinese character: 多様性, Hiragana: だいばーしてぃ, Katakana: ダイバーシティ, Alphabet: diversity} with same meaning. In token (19) in Japanese, {Katakana: ダイバーシティ} was divided into the sub words of “ダイバー (diver)” and “シティ(sity)”. So, the former “ダイバー (diver)” was focused as “diver” who dives as a sport, or who works or searches for things underwater using special breathing equipment, so this article may be predicted as SDG 14: marine life at 77.4%. This type of problem is not a matter of synonyms, but a language specific problem such as Chinese or Arabic, which pose challenges for morphological analysis.

### Visualization of SDGs nexus

**Fig. 3:**
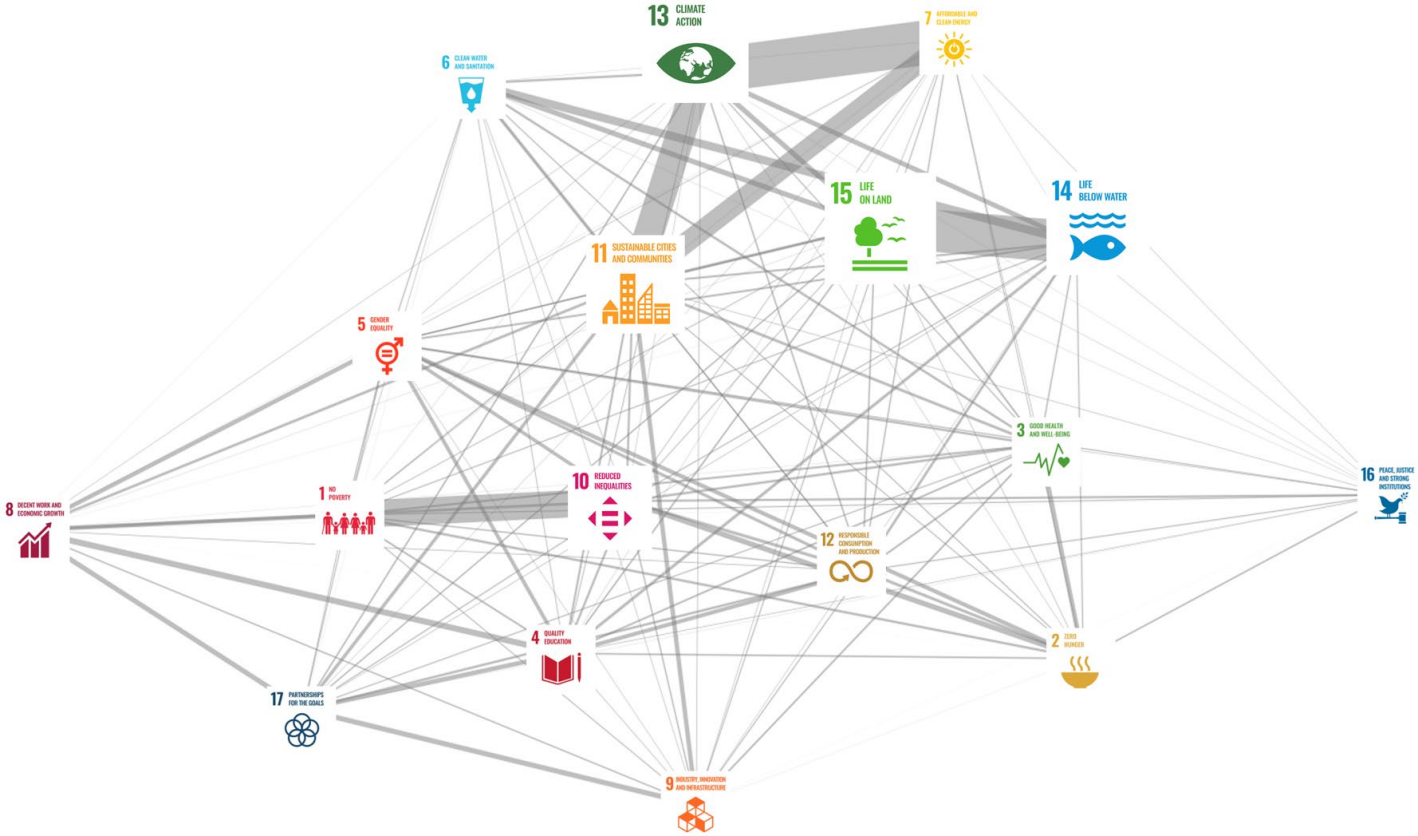
Visualization of SDGs nexus by analyzing the co-occurrences of predicted SDGs multi-labels with the Inventory of Business Indicators from SDG compass

Figure 3 is an SDGs nexus predicted by the model. First, the text classifier was applied to all indicators proposed in the Inventory of Business Indicators (*N* = 1429) in SDG compass (Global Reporting Initiative, UN Global Compact, and WBCSD 2015). All indicators in English were translated to Japanese manually and the translated indicator’s description was then input to the text classifier. SDGs related to each indicator were predicted in the multi-label format, then the predicted probability converted to 1 or 0 with a 50% threshold level to get the multihot vectors. The 17-dimentional multihot vectors, predicted as [0, 1, 1, 0, 1, 0, 0, 0, 0, 0, 0, 0, 0, 0, 0, 0, 0], can produce the co-occurrence relationship (e.g. SDG2 and 3, SDG2 and 5, SDG3 and 5 were co-occurred in this example). The co-occurrence among SDGs was analyzed and the SDGs nexus visualized. Nodes in Fig. 3 mean 17 SDGs and node sizes are proportional to the PageRank metrics (Brin and Page 1998), which is a score of the node’s influence within the network. Arcs connecting nodes in Fig. 3 are the co-occurrences between SDGs with the width proportional to the Jaccard-score (Jaccard 1912)—i.e., the closeness between the two goals. The libraries of scikit-learn (== 0.22.1) (Pedregosa et al. 2011) and network (== 2.5.1) (Hagberg et al. 2008) were used for the implementation of the Python environment.

Two major cores are observed in Fig. 3—the first core is SDG06 (clean water and sanitation), SDG07 (affordable and clean energy), SDG13 (climate action), SDG11 (sustainable city and community), SDG14 (marine life), and SDG15 (life on land) in the top right in Fig. 3. The second core is SDG01 (poverty eradication) and SDG10 (reduce inequality) in the bottom left in Fig. 3. In the microscopic view, the original indicators in the Inventory of Business Indicators have single-labels at the target level and are assumed to be for monitoring the performance of a single objective. However, the holistic coverage and nexus of SDGs predicted by the model instead suggested the nexus of human rights and equality, the empowerment of women and girls (International Labor Organization 2020; Alarcón and Cole 2019; Dhakal 2018; Mustafa 2019; Afenyo-Agbe and Adeola 2020), and the nexus of ecosystem management and climate action (Portner et al. 2021; Chiabai et al. 2018; Liu 2016; Sarkodie and Owusu 2017). From a macroscopic view these are the two major global challenges and their integration (Jackson and Decker Sparks 2020), and the model sheds light on a possibility to contribute to the global challenges from private sectors.

Currently, the three majors in the SDGs nexus research are: (1) statistical—to detect correlation or causality; (2) knowledge-driven—to infer causal chains based on real-world experience; (3) empirical—to analyze the process-based causal chain between SDGs through careful observation. This study proposes a fourth approach: multiple methods drawing on the network of SDGs by using natural language that focuses on semantics and infers the SDGs nexus by coupling data and knowledge. Especially the model can produce individual SDGs nexus inferences according to the stakeholder’s own data frame. The inferred SDGs nexus can be expected to promote the awareness and sharing of hidden interlinkages, and to potentially produce stakeholder’s collective works.

### Matchmaking of stakeholders

To create a matchmaking case, the model was applied to two municipalities in eastern and western Japan and 142 potential solutions from the private sector registered in (Cabinet Office Japan 2020). The sentences of the municipalities’ challenges and the solutions were converted to 768-dimensional vectors by the BERT model and the cosign similarity distance, which is a metric to evaluate the distance between vectors, calculated. The model then matched the challenges and solutions. Table 3 shows the summary: column 1 is the municipality’s name; column 2 is the sentences of the municipality’s challenge in original Japanese and translated English; columns 3 and 4 are the closest and farthest solutions from the private sector. The histogram on the left is the cosign similarity between the municipality and 142 solutions. Kakegawa, a city near metropolitan Tokyo, has a challenge in providing administrative services that allow citizens to move about as little as possible as mandated by the “new normal” imposed by COVID 19. The closest (highest cosign similarity) solution was a company providing audio and visual web content development, while the farthest (the lowest cosign similarity) solution was a biomaterial and bioenergy refinery company. Kishiwada City, which is famous for its “Danjiri festival” (Osaka Convention and Tourism; Bureau 2018), wants to promote and utilize its other natural and cultural resources. The company deemed to have the best solution packaged the regional resources, created a promotion strategy, and trained tourist guides. Lowest ranked was a human development company that offered teleworking support. Both cases make sense given the “needs and seeds”, so this appears to be rational matchmaking.

**Table 3:**
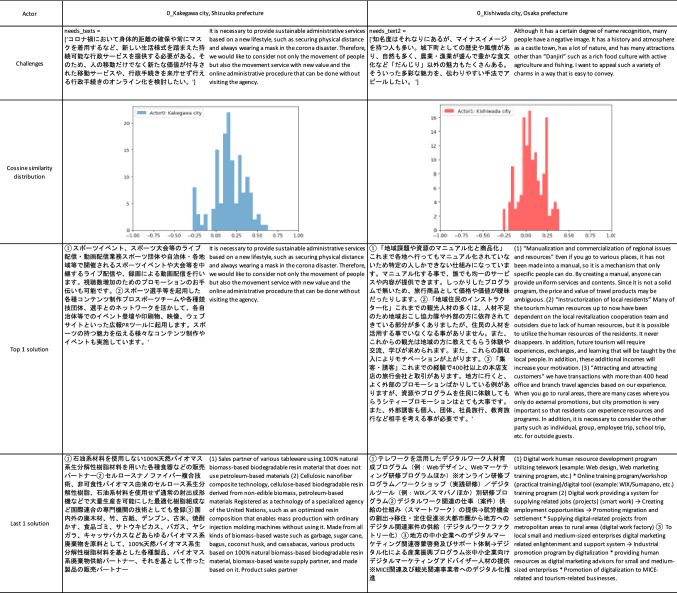
Cases of a match making between municipalities and private sector

**Fig. 4:**
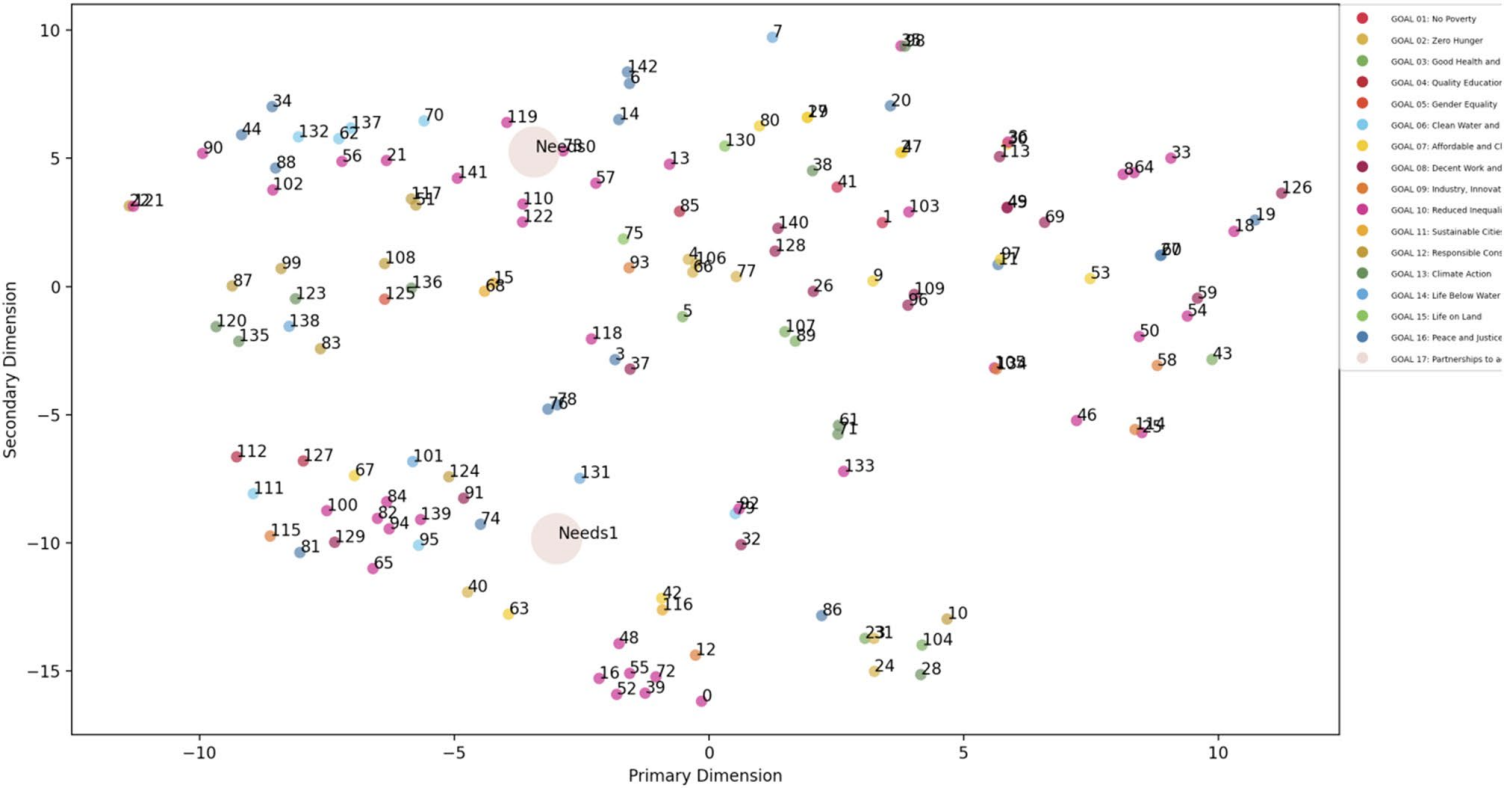
Matchmaking map by dimension reduction algorithm. Note: the small plots mean players which have potential solutions, and the large plots are players who have needs to be solved. The positions of the points were the two-dimensional coordinates obtained from original 768-dimensional vectors by dimension reduction using t-SNE. The color of points means the goal of SDGs that the players have the highest probability to be related to

Finally, Fig. 4 shows a visually supported map of stakeholder’s matchmaking. A dimension reduction algorithm was applied to convert all stakeholders’ 768-dimensional vectors to 2-dimensional vectors. The t-SNE (Maaten 2014) algorithm was used as the dimension reduction algorithm on scikit-learn library (== 0.22.1) on Python. The two large plots in Fig. 4 are the municipalities (Needs0 = Kakegawa and Needs1 = Kishiwada) and the small plots are the solutions. Each of the plots is embedded vector in the two-dimensional space and the color of the plot indicates the most suitable SDG as judged by the BERT model. Stakeholders can easily and globally see the potential candidates of matchmaking by referring to the semantic analysis and SDGs. Currently, in the (Cabinet Office Japan 2020) matchmaking event, a stakeholder shows some specific needs and the other stakeholders propose solutions, and (Cabinet Office Japan 2020) manually organizes a one-on-one session with empirical trial and error approach. This approach strongly depends on the organizer’s coordination resources. Our model can provide a readable map for all stakeholders and support to make the matchmaking process more transparent and reproducible. This function will be implemented our developing online platform, so we will validate the utility through practice with multi-stakeholders in future.

## Discussion

The improvement of model performance of the text classification and vectorization will contribute in fundamental ways to SDGs mapping and its application to nexus assessment and matchmaking tasks. There are many technical issues—for instance, the vocabulary and data size is small and the model needs to be much larger. However, four elements are essential to improve model development here.

### What is the accuracy?

The “accuracy” of the prediction itself is a difficulty. Indicators in SDG compass (Global Reporting Initiative, UN Global Compact, and WBCSD 2015) has a single-label format in the target level. As an evaluation of generalization performance, the model was tested to see if it could reproduce the single-label defined by the SDG compass. Table 4 shows the basic statistics of the corpus of indicators and the performance of the prediction. First, there was a significant difference in the basic text length between the training corpus (253.7 tokens/sentences) and indicators’ description in the SDG compass (30.3 tokens/sentences). A short sentence has few tokens or co-occurrences of tokens that characterize the meaning of sentences in the SDGs context. This tendency may affect the predictive reliability of the model, which was trained by long sentences. In fact, the mean predicted probability of indicators was 0.1 (S.D. 0.06) overall, which was a very conservative prediction. Thus, the score of recall, precision, and f1-score were quite low given a set threshold of 0.5 for the binarization. On the other hand, ROC/PR AUC, which are the metrics to evaluate the prediction performance (ranged from 0: poor to 1: good) by changing the threshold dynamically. The ROC-AUC had a fairly good outcome at 0.697 (S.E. 0.023), however, the PR-AUC had a bad outcome at 0.17 (S.E. 0.043). This result suggests that performance can change depending on which function we hope to the model prediction. Whether we require the model to predict both true positive and true negative or to actively predict only true positive gives a very different acceptance to the model performance.

**Table 4:** Corpus statistics and classification performance of Inventory of Business Indicators from SDG compass (Global Reporting Initiative, UN Global Compact, and WBCSD 2015)

SDGs	Number of indicator	Character/sentence	Token/sentence	Mean predicted probability	Recall	Precision	f1-score	ROC-AUC	PR-AUC
Overall	1479	49.4	30.3	0.10	0.26	0.19	0.21	0.70	0.17
Goal 01: No poverty	77	56.6	35.1	0.07	0.17	0.16	0.17	0.68	0.11
Goal 02: Zero hunger	43	49.1	29.7	0.05	0.16	0.15	0.16	0.57	0.05
Goal 03: Good health and well-being	96	55.7	34.2	0.06	0.15	0.23	0.18	0.64	0.15
Goal 04: Quality education	19	44.3	27.2	0.04	0.21	0.08	0.11	0.77	0.06
Goal 05: Gender equality	85	56.7	35.4	0.05	0.19	0.23	0.21	0.68	0.17
Goal 06: Clean water and sanitation	131	53.3	33.8	0.10	0.47	0.47	0.47	0.83	0.48
Goal 07: Affordable and clean energy	85	42.0	24.9	0.21	0.65	0.18	0.28	0.79	0.27
Goal 08: Decent work and economic growth	243	49.4	30.8	0.16	0.33	0.34	0.33	0.65	0.28
Goal 09: Industry, innovation and infrastructure	81	24.3	14.2	0.05	0.06	0.09	0.07	0.61	0.10
Goal 10: Reduced inequalities	53	37.9	22.8	0.06	0.13	0.09	0.10	0.62	0.03
Goal 11: Sustainable cities and communities	25	31.1	18.8	0.17	0.20	0.02	0.04	0.64	0.02
Goal 12: Responsible consumption and production	116	39.5	23.9	0.09	0.21	0.19	0.20	0.66	0.15
Goal 13: Climate action	96	55.1	33.4	0.25	0.55	0.15	0.24	0.76	0.18
Goal 14: Life below water	76	42.9	27.0	0.06	0.12	0.12	0.12	0.68	0.09
Goal 15: Life on land	131	65.4	40.5	0.10	0.36	0.37	0.36	0.78	0.32
Goal 16: Peace, justice and strong institutions	109	55.5	32.4	0.11	0.54	0.39	0.45	0.79	0.38
Goal 17: Partnerships for the goals	13	41.8	23.4	0.07	0.00	0.00	0.00	0.71	0.02

Note: recall, precision and f1-score were calculated by setting the threshold of the banalization of predicted probability in 0.5. ROC-AUC and PR-AUC denote the Area under Curve of ROC curve and Precision-Recall curve, which is a metric ranged 0 to 1 in machine learning field. 0 and means poor and excellent, respectively. ROC/PR-AUC in Overall means macro AUC, which is the mean of AUC by goal. All metrics were calculated by scikit-learn (== 0.22.1) (Pedregosa et al. 2011)

Noise in labeling by humans is also a significant issue. In the Inventory of Business Indicators from the SDG compass, “Average plant availability factor by energy source and by regulatory regime” is the indicator to evaluate target 1.4: “By 2030, ensure that all men and women, in particular the poor and the vulnerable, have equal rights to economic resources, as well as access to basic services, ownership and control over land and other forms of property, inheritance, natural resources, appropriate new technology and financial services, including microfinance.” This paper’s model predicted that this indicator belonged to GOAL 01 (no poverty): 0.001, GOAL 07 (affordable and clean energy): 0.796, GOAL 09 (industry, innovation and infrastructure): 0.312, GOAL 11 (sustainable cities and communities): 0.994, GOAL 13 (climate action): 0.974. Thus, while the human labeler might assume some kind of SDGs link between poverty and access to basic services with a human imagination, it appears that the model performed better. Moreover, the interpretation can change the contexts surrounding the stakeholder so right and wrong predictions are not entirely crucial. When it comes to that the SDGs, the important thing is to design the solutions with maturely considering synergies and trade-off, so the judgments of humans and AIs should complement each other.

### Single-label vs. multi-label

As stated in the preamble of the 2030 agenda (United Nations 2015),”SDGs are integrated and indivisible and balance the three dimensions of sustainable development: the economic, social, and environmental…”. Therefore, SDGs mapping should be a multi-label task. (Zhang et al. 2020a, b), using a corpus (*N* = 606) with single label tried to train both conventional feature-based and deep learning-based machine learning algorithms (Naïve Bayes, Support Vector Machine, Logistic regression, Convolutional Neural Network, Long Short-Term Memory, ELMo, BERT). None of the models could achieve more than a 0.1 in f1-score, irrespective of the type of algorithm. We also checked and confirmed the reproducibility of this tendency by training the model using only single label corpus. The concept of “decarbonization” is obviously related to both SDG 07 (affordable and clean energy) and SDG13 (climate action)—however, in the single-label classification task, “decarbonization” can be linked only to SDG 07 or to SDG 13 or neither. This restriction severely affects the training of the source-target attention layers in the BERT model. Given this, we are convinced that the text classification task in the SDGs field definitely requires a multi-label data frame for both model training and the SDGs nexus.

### Language dependency of accuracy

Our text classification model training displays high performance. (Guisiano and Chiky 2021) also conducted a multi-label classification task with the augmented SDGs documents in English and achieved an accuracy rate of over 0.90, an excellent performance. However, each language has separate difficulties in collecting documents, preprocessing corpus, so there is little meaning in comparing accuracy among languages. (Zhang et al. 2020a, b) used ALBERT, a simpler version of BERT (Lan et al. 2020), and developed a system to infer the nexus between 4005 of SDGs activities in Japanese and achieved 0.7 accuracy. They implied that the sentences that included multiple languages make classification difficult. As shown in an example of “biodiversity” in the result of this research, Japanese uses a lot of English so this study’s corpus included many mixed sentences in Japanese and English. From a technical aspect, the Tohoku-Japanese pretrained model originally used the Japanese Wikipedia database, and this model divides English words into all alphabet with WordPiece algorithms, such as “SDGs”—> {“S,” “D,” “G,” “s”}. It goes without saying that the BERT model must learn the relationship and the order of all of the original meaning in tokens may be lost in the self-attention processing. As (Amin et al. 2019) pointed out, better cross-lingual and cross-domain embedding alignment methods that can transferred effectively will encourage research. And these works are not competitive but collective as described below.

### Gigantic global model and indigenous local model

On this occasion, we attempted to build a text classification system localized in Japanese. However, SDGs has a globally universal agenda, which must be sharable in any language. There are two alternatives. One is to develop a universal semantic processing model based on an ultra-giant model such as GPT-3 (Generative Pre-trained Transformer 3) (Brown et al. 2020) and fine-tuned through a gigantic corpus comprising SDGs knowledge from all over the world translated in a universal language. The global SDGs projects, such as AI for Good project (International Telecommunication Union 2017), are expected to meet this challenge. The other alternative, as the history of the Local Agenda 21 (United Nations 1992) and the promotion of the Local 2030 (United Nations 2017) suggests, is that the essentials of SDGs achievement may be locally driven, based on an ensemble approach for the globally thinking and locally acting stakeholders. Each regional and local community, including languages archived in the Atlas of the World’s Languages in Danger (Moseley 2010), develops their SDGs semantic models in their original language, and the models utilizes indigenous local knowledge and creates an ensemble wisdom under the global collaboration.

## Conclusion

This study established an SDGs corpus in Japanese and extracted the sentences related to SDGs with multi-label annotation. The BERT, a state-of-the-art model for natural language processing, was trained with this SDGs corpus to build a text classifier model that can identify the SDGs related to the input sentences and also vectorize the semantics. By using the model, a nexus among SDGs was predicted from a representative indicator database and potential applicability to matchmake the stakeholders for SDGs collaboration. Finally, the model had a generally good performance and further development points were discussed, such as the accuracy improvement and a globalization and localization strategy.

For future exploration, we will attempt to establish corpora in the six official languages of the United Nations and verify the interoperability of corpora for model learning across languages and the possibility of diverting the trained models to other languages. And as a further trial, we will also attempt to design a generative model which can convert inputted normal sentences to edited sentences that were translated in the SDGs context. This will be supported in multiple languages and the corpus and models will be implemented on Platform Clover for global collaborations.

## Supplementary Information

Below is the link to the electronic supplementary material.

Supplementary file1 (XLSX 17 KB)
